# Retraction: Down-regulation of microRNA-138 improves immunologic function via negatively targeting p53 by regulating liver macrophage in mice with acute liver failure

**DOI:** 10.1042/BSR-2019-0763_RET

**Published:** 2023-06-15

**Keywords:** Acute liver failure, Immune function, Macrophage, microRNA-138, P53

This article is being retracted from *Bioscience Reports* at the request of the Editor-in-Chief and the Editorial Board following receipt of a notification from a reader, alerting the Editorial Board to cell-count distribution graphs that do not conform to the expected results for an experiment of this type.

The authors were asked to provide the raw data in the form of the output from the flow cytometer, including the specific datapoints used to build the graphs. The authors provided the Editorial Board with PDF scatterplots but were unable to provide the specific data points, stating that this data was not saved. Given the lack of raw data, and that doubt remains over the validity of the graphs, the Editorial Board stands by the decision to retract. The authors agree to the retraction.

